# Bioactive potential of Bio-C Temp demonstrated by systemic mineralization markers and immunoexpression of bone proteins in the rat connective tissue

**DOI:** 10.1007/s10856-024-06781-3

**Published:** 2024-02-14

**Authors:** Camila Soares Lopes, Mateus Machado Delfino, Mário Tanomaru-Filho, Estela Sasso-Cerri, Juliane Maria Guerreiro-Tanomaru, Paulo Sérgio Cerri

**Affiliations:** 1 Department of Restorative Dentistry, Dental School, São Paulo State University (Unesp), Araraquara, SP Brazil; 2 Laboratory of Histology and Embryology, Department of Morphology, Genetics, Orthodontics and Pediatric Dentistry, Dental School, São Paulo State University (Unesp), Araraquara, SP Brazil

## Abstract

Intracanal medications are used in endodontic treatment due to their antibacterial activity and ability to induce the periapical repair. Among the intracanal medications, the Calen (CAL; SS. White, Brazil) is a calcium hydroxide-based medication that provides an alkaline pH and releases calcium, exerting an antimicrobial activity. Bio-C Temp (BIO; Angelus, Brazil), a ready-to-use bioceramic intracanal medication, was designed to stimulate the mineralized tissues formation. Here, we investigated the bioactive potential of BIO in comparison to the CAL in the rat subcutaneous. Polyethylene tubes filled with medications, and empty tubes (control group, CG) were implanted in the subcutaneous tissue of rats. After 7, 15, 30 and 60 days, the blood was collected for calcium (Ca^+2^) and alkaline phosphatase (ALP) measurement, and the capsules around the implants were processed for morphological analyses. The data were submitted to two-way ANOVA and Tukey test (p < 0.05). At 7, 15 and 30 days, the ALP level was grater in BIO and CAL than in CG (p < 0.0001). At 7 and 15 days, greater Ca^+2^ level was seen in the serum of CAL samples. From 7 to 60 days, an increase in the number of fibroblasts, osteocalcin- and osteopontin-immunolabelled cells was observed in BIO and CAL groups (p < 0.0001). In all periods, BIO and CAL specimens showed von Kossa-positive structures. Moreover, ultrastructural analysis revealed globules of mineralization in the capsules around the BIO and CAL specimens. Thus Bio-C Temp caused an increase in the ALP, osteocalcin and osteopontin, which may have allowed the formation of calcite, suggesting bioactive potential.

**Graphical Abstract:**

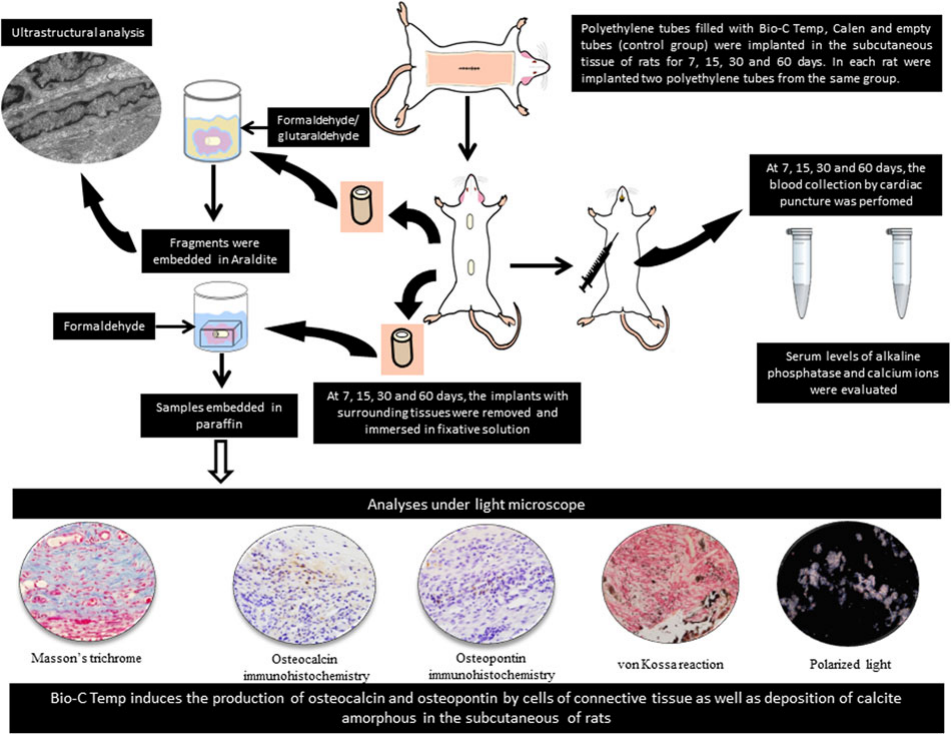

## Introduction

Calcium hydroxide is recommended during endodontic treatment due to its antimicrobial action and biological properties, such as its ability to induce deposition of mineralized tissue [1, 2]. It has been demonstrated that calcium hydroxide induces mineralization due to its ionic dissociation giving rise to calcium and hydroxyl ions in an aqueous medium [3] providing an alkaline environment - that favors repair tissue [4]. Alkaline environment favors the calcification [3], since neutralizes lactic acid from osteoclasts and activates alkaline phosphatase (ALP), an enzyme that has an important role in the formation of hard tissues [1]. Calcium ions also play an essential role in mineralization, stimulating the expression of the fibronectin gene [4].

Previously to the use of a filling material, periodic changes of calcium hydroxide-based intracanal medicament are used [5] to induce the formation and repair of mineralized tissue [6]. Among the indications, these intracanal medications are used for treatment of dental trauma [5, 7], root perforations [6], root resorption, apexification and as a capping agent during pulpotomy [2]. However, it has been suggested that long-term exposure to calcium hydroxide-based medications induces collagen degradation [5] and, hence, weakening the root dentine [8]. Moreover, the calcium hydroxide-based pastes have low radiopacity and flow capacity, which difficult their insertion into the root canal [9].

Bioceramic endodontic materials have stood out for being bioactive [10], due to the interaction of the material with dentine, forming a mineralized intermediate zone in the presence of moisture [11]. Furthermore, the bioceramic materials modulate the response of host cells favoring the bone repair, since favors the osteoblasts survival and differentiation, cells actively involved in the periapical repair [12] enabling the formation of mineralized tissue [13]. Bioceramic endodontic materials are biocompatible [14, 15], non-cytotoxic [16] and have satisfactory physico-chemical properties [16–18]. There is currently a growing interest in the development of new materials based on bioactive calcium silicate [19].

Bio-C Temp (Angelus Indústria de Produtos Odontológicos, Londrina, PR, Brazil) is a ready-to-use calcium silicate-based paste, which contains calcium silicates associated with calcium tungstate and titanium oxide radiopacifiers, calcium aluminate, calcium oxide and base resin as a vehicle that does not allow hydration and setting of the material. Bio-C Temp is an intracanal medication that eliminates the need for new applications, constituting an advantage over calcium hydroxide-based medications that require frequent changes [20]. According to the manufacturer, this material can be used in teeth with treatment of incomplete root formation, perforations, external and internal root resorption, previously to the use of repair and filling materials. Moreover, Bio-C Temp has been suggested in pulpotomy cases for the induction of a calcified barrier [21] and treatment of and endodontic regeneration [22, 23].

Human dental pulp cells (hDPCs) cultured with diluted extracts of Bio-C Temp suggested that this intracanal medication presents a dose and time cytotoxic dependent [19]. After 60 days of implantation in the subcutaneous connective tissue, the Bio-C Temp was biocompatible as well as did not cause changes in liver enzymes [24]. Furthermore, alkaline phosphatase activity and deposition of mineralized nodules have been shown in human osteoblast-like cell line (Saos-2) culture containing diluted extracts of Bio-C Temp [23], suggesting that this medication could present a bioactive potential. However, Bio-C Temp implanted into subcutaneous tissues for 28 days promoted a lower amount of calcium and phosphorous deposits than the Vitapex, a calcium hydroxide intracanal medication [25].

Bio-C Temp intracanal medication provides an alkaline pH to the microenvironment and releases calcium ions when immersed in solution [19, 21], essential requirements in inducing the differentiation of cells that produce organic matrix components of mineralized tissues. Materials that promote increase in osteogenic response are of great value for endodontic therapy [12]. Extracellular matrix proteins of mineralized tissues, such as osteocalcin (OCN) and osteopontin (OPN) evaluated by immunohistochemistry reactions, are widely used to evaluate the bioactive potential of endodontic materials [14, 26–28]. In addition, inorganic constituents of mineralized tissues including calcium (Ca^+2^), the ALP, an enzyme involved in the mineralization, can be measured in the serum in order to investigate whether the endodontic materials cause changes in these parameters [27, 29].

Therefore, in the present study, we evaluated the bioactive potential in vivo of an intracanal medication of calcium silicate, Bio-C Temp, in comparison with calcium hydroxide-based intracanal medication (Calen, SS. White Art. Dent. Ltda, RJ, Brazil). For this purpose, the concentrations of Ca^+2^ and ALP in the serum were measured at 7, 15, 30 and 60 days after implantation of Bio-C Temp in the subcutaneous tissues of rats. Moreover, immunoexpression of OCN and OPN, typical proteins of mineralized tissues, and deposition of calcite in the capsules around the implants were also evaluated.

## Material and methods

### Experimental procedures

This research (protocol # 22/2018) was approved by the Ethical Committee for Animal Research of Araraquara Dental School (São Paulo State University–UNESP, Brazil) in compliance with Brazilian national law on animal use. The study was carried out in accordance with the US National Institute of Health Guide for the Care and Use of Laboratory Animals (NIH Publications n° 80-23, 1996). The experiment and analyses method were conducted in accordance with ARRIVE guidelines 2.0 (Animal Research: Reporting of In Vivo Experiments). Sixty Holtzman (*Rattus norvegicus albinus*) adult male rats, weighing 250–280 g were randomly distributed into three groups (n = 20 per group): BIO (BIO-C TEMP Group, Angelus, Londrina, Brazil), CAL (Calen Group, SS. White Art. Dent. Ltda, RJ, Brazil) and CG (control group, empty polyethylene tubes). Intracanal medications and their chemical composition are described in Table 1 (Supplement).

The polyethylene tubes (Embramed Indústria Comércio, São Paulo, SP, Brazil) with 10.0 mm length and 1.6 mm diameter, previously sterilized with ethylene oxide, were filled with the materials or kept empty (CG) and implanted into the subcutaneous connective tissue. Previously, the animals were anaesthetized with an intraperitoneal injection of ketamine hydrochloride (80 mg/kg of body weight) combined with xylazine hydrochloride (8 mg/kg of body weight). After trichotomy and disinfection with 5% iodine solution, a 2.0 cm-long incision was made in a head-to-tail orientation using a n° 15 scalpel (Fibra Cirúrgica, Joinvile, SC, Brazil), and the polyethylene tube was placed into the subcutaneous pocket.

After 7, 15, 30 and 60 days of implantation, the animals were anaesthetized, as previously described, and cardiac puncture of the left ventricle was performed with a vacuum tube (Vacuette®, Greiner Bio-One Brasil Produtos Médicos Hospitalares Ltda., Americana, SP, Brazil) with a needle (Med Goldman Indústria e Comércio Ltda., Manaus, AM, Brazil) and connected to a BD Vacutainer® adapter (Becton Dickinson Indústrias Cirúrgicas Ltda.,Curitiba, PR, Brazil). After blood collection, the animals were killed with anesthetic overdose and the implants and surrounding tissues were removed. The specimens were fixed for 48 h in 4% formaldehyde buffered with 0.1 M sodium phosphate at pH 7.2, and the specimens were processed for paraffin embedding. Non-serial Section (6 µm thick) were adhered to slides previously treated with 4% silane (Sigma-Aldrich). The sections were subjected to immunohistochemistry reactions for detection of OCN and OPN. Other non-serial sections were submitted to the von Kossa histochemical reaction to evaluate the calcium deposits in the capsules and Masson’s trichrome staining was used for the quantification of fibroblasts [30].

### Concentration of Ca^+2^ and ALP in the serum

After blood collection (as described above) and clot formation, the blood was centrifuged (Excelsa® II 206 BL; Fanem Ltda., Guarulhos, SP, Brazil) at 3500 rpm for 10 min and the serum was stored at −20 °C. The calcium and ALP concentrations were determined using the Calcium Arsenazo III assay kit (Beckman Coulter, Indianapolis, Indiana, USA) and Alkaline Phosphatase (Beckman Coulter), respectively. The sample absorbance was read on a spectrophotometer (Hitachi, model U 1100, Tokyo, Japan). The absorbance of calcium was determined at 660/700 nm, phosphorus at 340 nm and ALP at 410/480 nm. The experiments were carried out in triplicate and the averages were calculated.

### Numerical density of fibroblasts

The number of fibroblasts was estimated from three non-serial sections stained with Masson’s trichrome. The images of the capsules adjacent to the implants were captured with a camera (DP-71, Olympus - Japan) attached to a light microscope (Olympus, model BX-51), using the x40 objective (x695 final magnification). The fibroblasts identified as fusiform or elliptical shape cells were estimated in a standardized field (0.09 mm^2^) using an image analysis system (Image-Pro Express Olympus). In each specimen, the fibroblasts were counted in a total standardized area of 0.27 mm^2^, and the number of fibroblasts per mm^2^ was estimated [30].

### Immunohistochemical detection of OCN and OPN

Deparaffinized sections were immersed in 0.001 M sodium citrate buffer at pH 6.0 and heated in a microwave oven at 96–98 °C for 20 min. After cooling, the slides were washed in 0.01 M sodium phosphate buffer (PBS) for 15 min and immersed for 30 min in 5% hydrogen peroxide to inactive the endogenous peroxidase. The sections were incubated for 20 min with 2% bovine serum albumin (Sigma-Aldrich Co., Saint Louis, Missouri, USA) at room temperature. Sections were then incubated for 16 h with mouse anti-osteocalcin monoclonal antibody (Sigma-Aldrich Co., Saint Louis, Missouri, USA; code SAB1306277; diluted at 1:300) or mouse anti-osteopontin monoclonal antibody (Abcam Inc., Cambridge, MA, USA; code: ab 166709; diluted at 1:100) in a humid chamber at 4 °C. After washings, the sections were incubated for 1 h with labeled polymer-HRP (EnVision + Dual Link System-HRP, Dako Inc., Carpinteria, CA, USA; K4061) at room temperature. The peroxidase activity was revealed by 3,3’-diaminobenzidine chromogen (ImmPACTTM DAB substrate, Vector Laboratories, Burlingame, CA, USA) for 3 min. The slides were washed with distilled water and, subsequently, the sections were counterstained with haematoxylin. In negative controls, the sections were incubated with non-immune serum.

The number of OCN- and OPN-immunolabelled cells was quantified in the capsules of all the animals. Using a light microscope (Olympus, BX-51) and an image analysis system (Image-Pro Express Olympus), the number of immunolabelled cells was estimated in a standardized area (0.09 mm^2^) of the capsule in close juxtaposition to the opening of implanted tube [14].

### von Kossa histochemical reaction and analysis under polarized light

The deparaffinized sections were incubated in an aqueous solution containing 5% silver nitrate, under the action of an incandescent light (100 watts) for 1 h. After incubation, the sections were washed quickly in distilled water and, subsequently, the sections were immersed in 5% sodium hyposulfite for 5 min. Then, the sections were washed in running water and for 5 min in distilled water. After washing, the sections were submitted to the picrosirius-red method, washed, dehydrated and mounted in resinous medium (Permount®, Fisher Scientific, New Jersey, USA) for analysis under a light microscope.

Dewaxed sections of all specimens were mounted in resinous medium (Permount®, Fisher Scientific, New Jersey, USA). These unstained sections were analyzed under polarized light to assess the presence of birefringent structures in the capsules [15, 31–33].

### Ultrastructural analysis

The bioactive potential of medications was also evaluated under transmission electron microscopy analysis. This analysis was performed in three specimens of each group at 60 days. The implants surrounded by tissues were fixed for 24 h in a solution of 4% glutaraldehyde and 4% formaldehyde buffered at pH 7.2 with 0.1 M sodium cacodylate [31]. After washings in 0.1 M sodium cacodylate and removing of the polyethylene tubes, small fragments of capsules were post-fixed with 1% osmium tetroxide for 1 h, washed in distilled water and the specimens were immersed in 2% aqueous uranyl acetate during 2 h. After dehydration, the specimens were treated with propylene oxide and embedded in Araldite^®^ (Electron Microscopy Sciences). The regions of capsules were selected from semithin sections stained with 1% toluidine blue and the ultrathin sections obtained with diamond knife were collected onto grids. The ultrathin sections were stained with 2% uranyl acetate and lead citrate solution. The ultrathin sections were examined using a transmission electron microscope (Tecnai G2 Spirit, FEI Company).

### Statistical analysis

The statistical analyses were performed using the GraphPad Prism 6.01 program (GraphPad Software, Inc., La Jolla, CA, United States). The data were submitted to two-way ANOVA analysis of variance, followed by the Tukey test (p < 0.05). All data were presented as mean ± standard deviation.

## Results

### Concentration of calcium and ALP in the serum

**Fig. 1:**
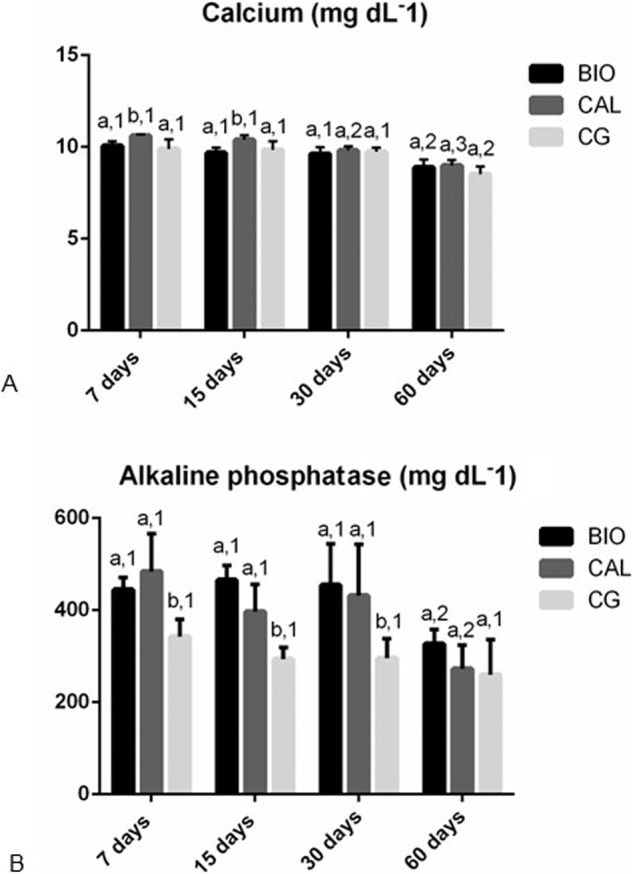
Graphics showing the values (expressed as mean ± standard deviation) of the concentration of calcium (**A**), and alkaline phosphatase (**B**) in the serum of the Bio-C Temp (BIO), Calen (CAL) and CG specimens at 7, 15, 30 and 60 days. In each period, the comparison among the groups is indicated by superscript letters; different letters = significant difference. The superscript numbers indicate the analysis of each group over time; different numbers = significant difference. Tukey’s test (p ≤ 0.05)

In all periods, the serum Ca^+2^ concentration in BIO group was similar to that of CG (p > 0.05). In contrast, the serum Ca^+2^ levels were greater in CAL group than in BIO and CG groups at 7 and 15 days. At 30 and 60 days, no significant difference among BIO, CAL and CG specimens was observed in the Ca^+2^ concentration (p < 0.0001). From 7 to 60 days, the Ca^+2^ level reduced significantly in all groups (p < 0.0001) (Fig. 1A).

According to Fig. 1B, the serum ALP levels in the BIO and CAL groups were significantly higher than in the CG specimens at 7, 15 and 30 days (p < 0.0001). Moreover, there was no significant difference in the ALP concentration between BIO and CAL medications (p > 0.05). At 60 days, no significant difference was found among the BIO, CAL and CG groups (p > 0.05). From 30 to 60 days, the serum ALP levels decreased significantly in the BIO and CAL groups (p < 0.0001) while no significant difference in the ALP concentration was observed in the CG specimens over time (p > 0.05).

### Histological features and number of fibroblasts in the capsules

**Fig. 2:**
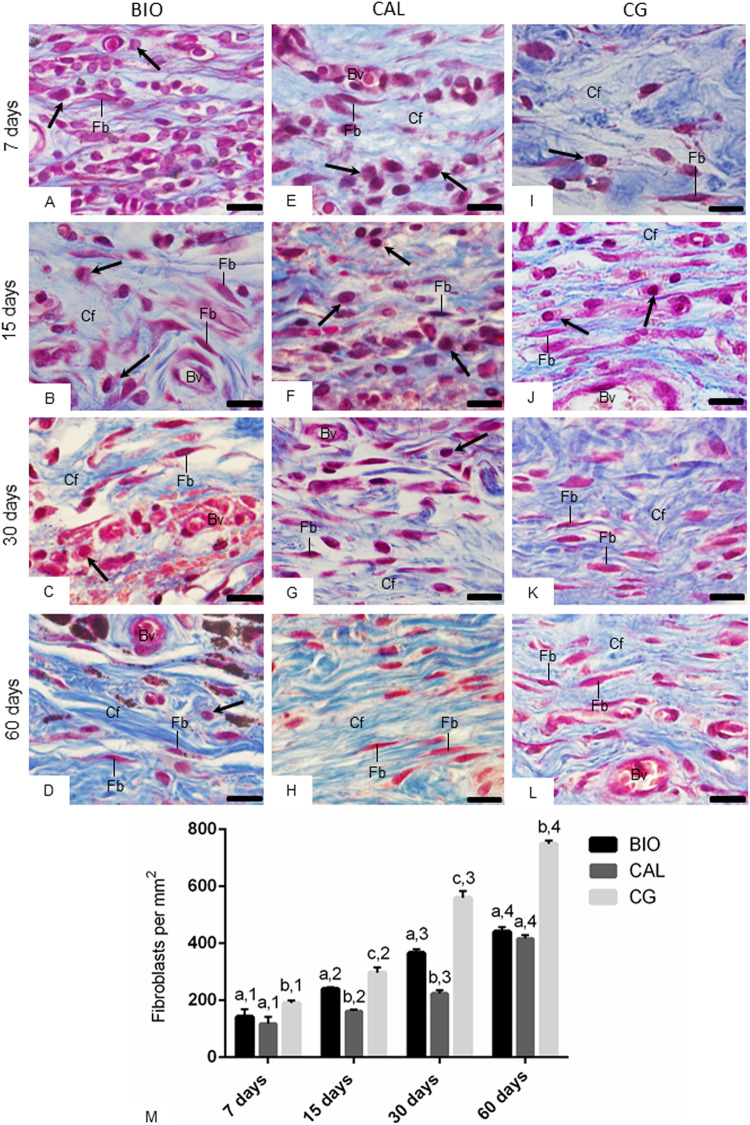
Photomicrographs showing portions of the capsules adjacent to the opening of the implanted tubes of the BIO (**A**–**D**), CAL (**E**–**H**) and CG (**I**–**L**) groups show an increase in fibroblasts (Fb) and collagen fibers (in blue) over time. Arrows, inflammatory cells; Bv, blood vessels; Cf, collagen fibers. Masson’s trichrome. Bars: 18 μm. Figure 2**M** Graphic showing the number of fibroblasts per mm^2^ (expressed as mean ± standard deviation) from BIO, CAL and CG groups at 7, 15, 30 and 60 days. In each period, the comparison among the groups is indicated by superscript letters; different letters = significant difference. The superscript numbers indicate the analysis of each group over time; different numbers = significant difference. Tukey’s test (p ≤ 0.05)

The analysis of sections stained with Masson’s trichrome (Fig. 2A–L) showed some changes in the cell population and extracellular matrix components of capsules adjacent implants over time. After 7 days, the capsules contained few fibroblasts among many inflammatory cells (Fig. 2A, E, I). Over the course of 15 and 30 days, a decrease in the number of inflammatory cells in parallel to an increase in the number of fibroblasts and bundles of collagen fibers, stained in blue, were observed (Fig. 2B, C, F, G, J, K). At 60 days (Fig. 2D, H, L), the capsules exhibited mainly fibroblasts intermingled with an enhanced content of thick collagen bundles compared to those seen at 7 days.

According to Fig. 2M, the quantitative analysis of the capsules of all groups revealed a gradual and significant increase in the numerical density of fibroblasts (p < 0.0001). At 7 and 60 days, there was no significant difference in the number of fibroblasts between BIO and CAL groups (p = 0.0703). In contrast, at 15 and 30 days, the number of fibroblasts was greater in BIO group than in CAL specimens (p < 0.0001). In all time points, the greatest values of fibroblasts were observed in the CG specimens (p < 0.0001).

### OCN and OPN immunoexpression in capsules

**Fig. 3:**
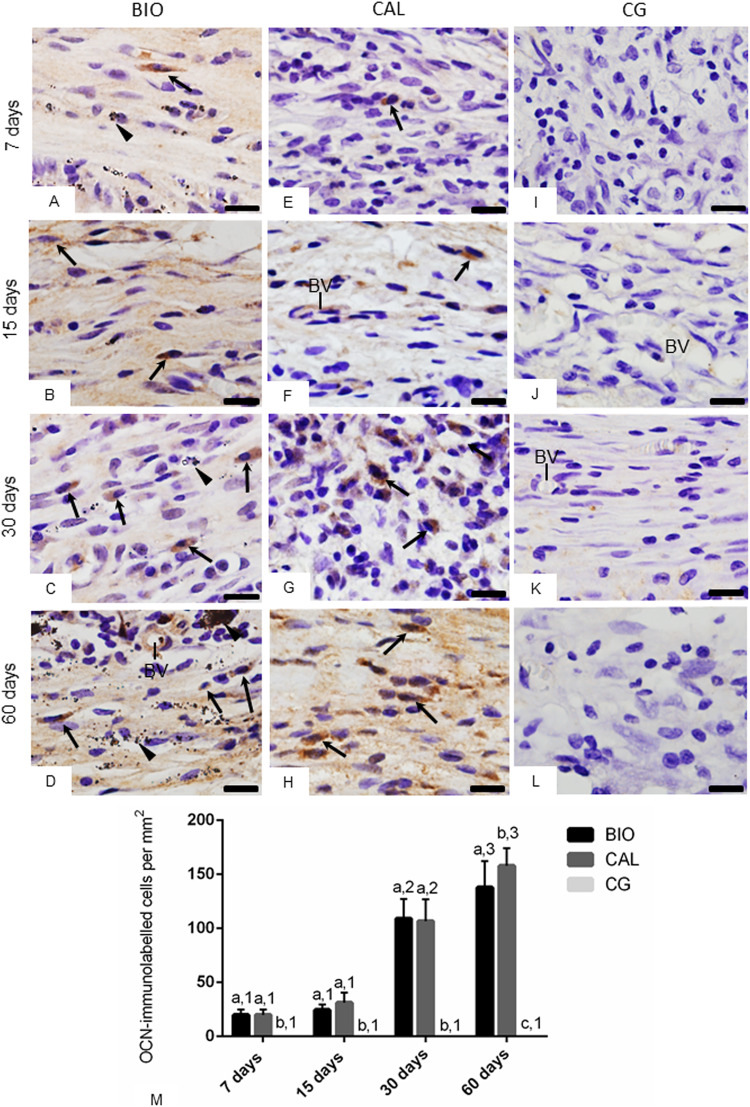
**A–L** Photomicrographs showing portions of sections submitted to immunohistochemistry for detection of OCN (in brown colour) and counterstained with haematoxylin. In BIO (**A**–**D**) and CAL (**E**–**H**) some elliptical and/or fusiform cells exhibit immunolabelling in their cytoplasm (arrows). None immunolabelled cell is observed in the CG specimens (**I**–**L**). Arrowhead: material particles; BV, blood vessels. Bars: 18 μm. Figure 3**M**—Graphic showing the number of OCN-immunolabelled cells per mm^2^ (expressed as mean ± standard deviation) from BIO, CAL and CG groups at 7, 15, 30 and 60 days. In each period, the comparison among the groups is indicated by superscript letters; different letters = significant difference. The superscript numbers indicate the analysis of each group over time; different numbers = significant difference. Tukey’s test (p ≤ 0.05)

**Fig. 4:**
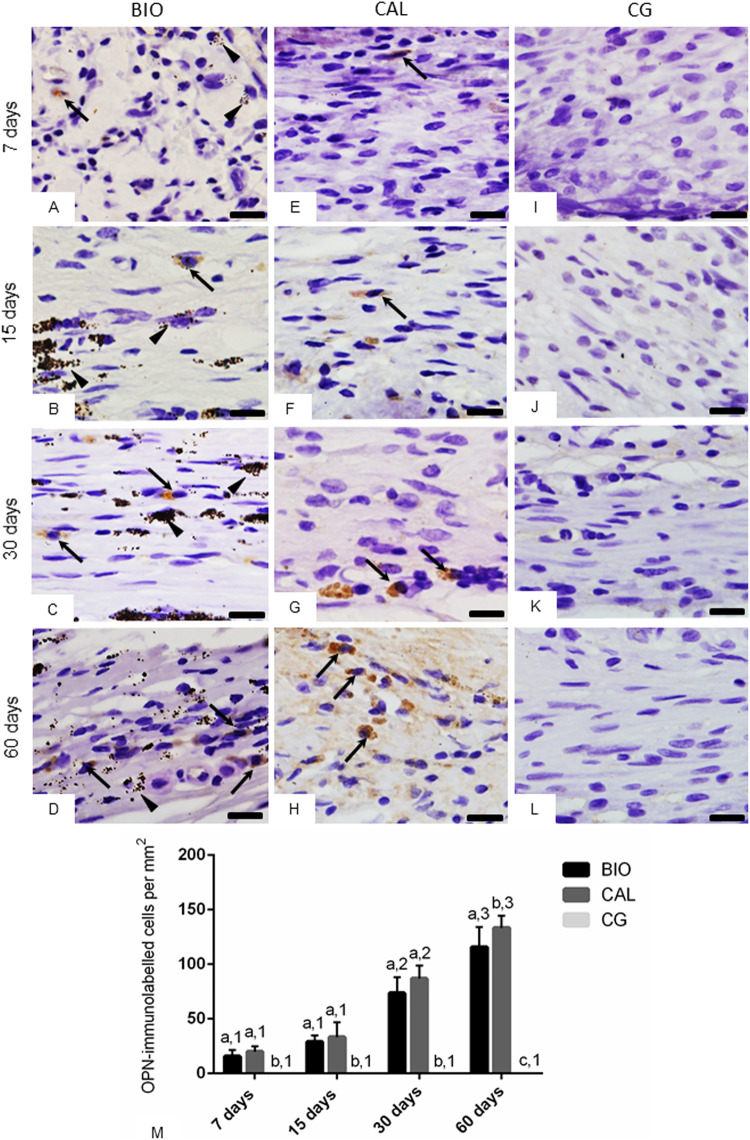
**A–L** Photomicrographs showing portions of the sections submitted to immunohistochemistry for detection of OPN (in brown colour) and counterstained with haematoxylin. At 7 and 15 days, few immunolabelled cells (arrows), mainly fibroblasts, are observed in the capsules of BIO (**A**, **B**) and CAL (**E**, **F**). At 30 and 60 days, several strongly immunolabelled cells (arrows) are present in the capsules of BIO (**C**, **D**) and CAL (**G**, **H**) groups. Note absence of immunolabelled cells in the capsules of CG specimens (**I**–**L**). Arrowhead: material particles. Bars: 18 μm. Figure 4**M** Graphic showing the number of OPN-immunolabelled cells per mm^2^ (expressed as mean ± standard deviation) from BIO, CAL and CG groups at 7, 15, 30 and 60 days. In each period, the comparison among the groups is indicated by superscript letters; different letters = significant difference. The superscript numbers indicate the analysis of each group over time; different numbers = significant difference. Tukey’s test (p ≤ 0.05)

The sections submitted to immunohistochemical reactions for detection of OCN (Fig. 3A–H) and OPN (Fig. 4A–H) showed immunostained cells (brown/yellow colour) in the capsules of BIO and CAL specimens, in all periods. The capsules around the medications contained some elliptical and round shaped cells with strong OCN (Fig. 3A–H) and OPN (Fig. 4A–H) immunolabelling in their cytoplasm. OCN- and OPN-immunolabelled cells were not observed in the CG specimens (Figs. 3I–L and 4I–L, respectively).

The quantitative analysis of the number of OCN- and OPN-immunopositive cells (Figs. 3M, 4M, respectively) revealed that there were no significant differences between the BIO and CAL groups at 7, 15 and 30 days (p > 0.05). However, at 60 days the number of OCN- and OPN-immunolabelled cells was greater in the CAL than in BIO specimens (p < 0.0001). From 7 to 15 days, no significant difference was observed in the number of OCN- and OPN-immunolabelled cells in the BIO and CAL specimens, but the number of immunolabelled cells increased significantly from 15 to 30 days (p < 0.0001) and from 30 to 60 days (p < 0.0001) in both groups. In contrast, no significant different was detected in the CG specimens over time.

### von Kossa histochemical reaction and analysis of unstained sections

**Fig. 5:**
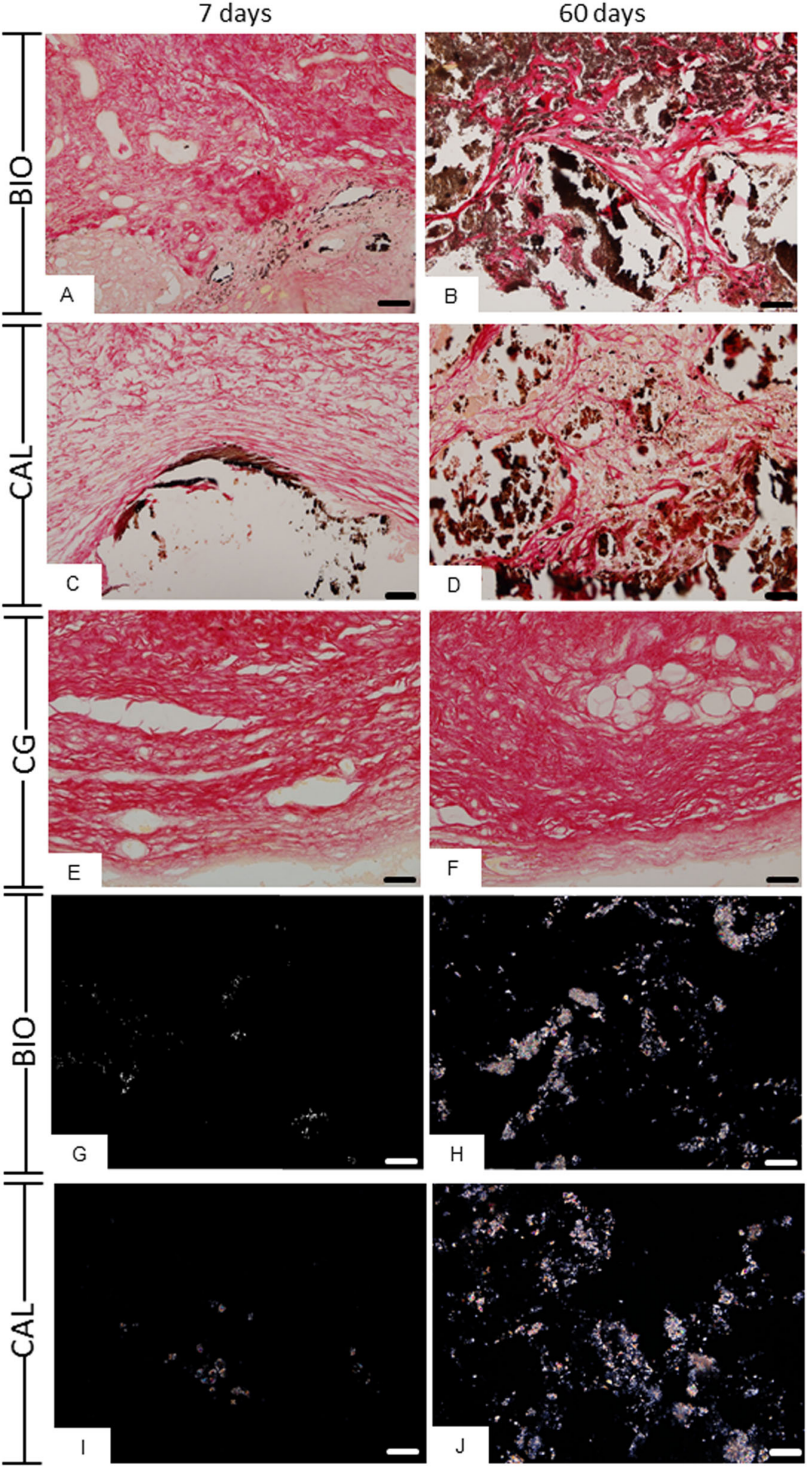
Photomicrographs showing portions of capsules adjacent to the opening of the tubes implanted in the subcutaneous for 7 (**A**, **C**, **E**, **G**, **I**) and 60 days (**B**, **D**, **F**, **H**, **J**). **A–F** Sections submitted to the von Kossa reaction and counterstained with picrosirius-red. von Kossa-positive structures (black colour) are observed dispersed in the capsules of BIO (**A**, **B**) and CAL (**C**, **D**) specimens. In (**E**, **F**), no positive structures are observed in CG. Figure 5**G–J** Photomicrographs showing unstained sections analyzed under polarized light. Note the presence of birefringent structures in the BIO and CAL groups at 7 and 60 days. Bars: 52 μm

The sections of BIO (Fig. 5A, B) and CAL (Fig. 5C, D) implants submitted to the von Kossa histochemical method revealed positive structures (in black/brown colour) in all periods. von Kossa-positive deposits were observed dispersed between cells and collagen fibers as well as in the capsule surface (medication/connective tissue interface). von Kossa-positive deposits were not observed in the capsules of CG specimens (Fig. 5E, F).

The unstained sections analyzed under polarized light revealed birefringent structures (Fig. 5G–J) in similar regions of those observed in von Kossa reactions (Fig. 5A–D). Moreover, no birefringent structure was seen in the CG specimens (data no shown).

### Transmission electron microscopy analysis

**Fig. 6:**
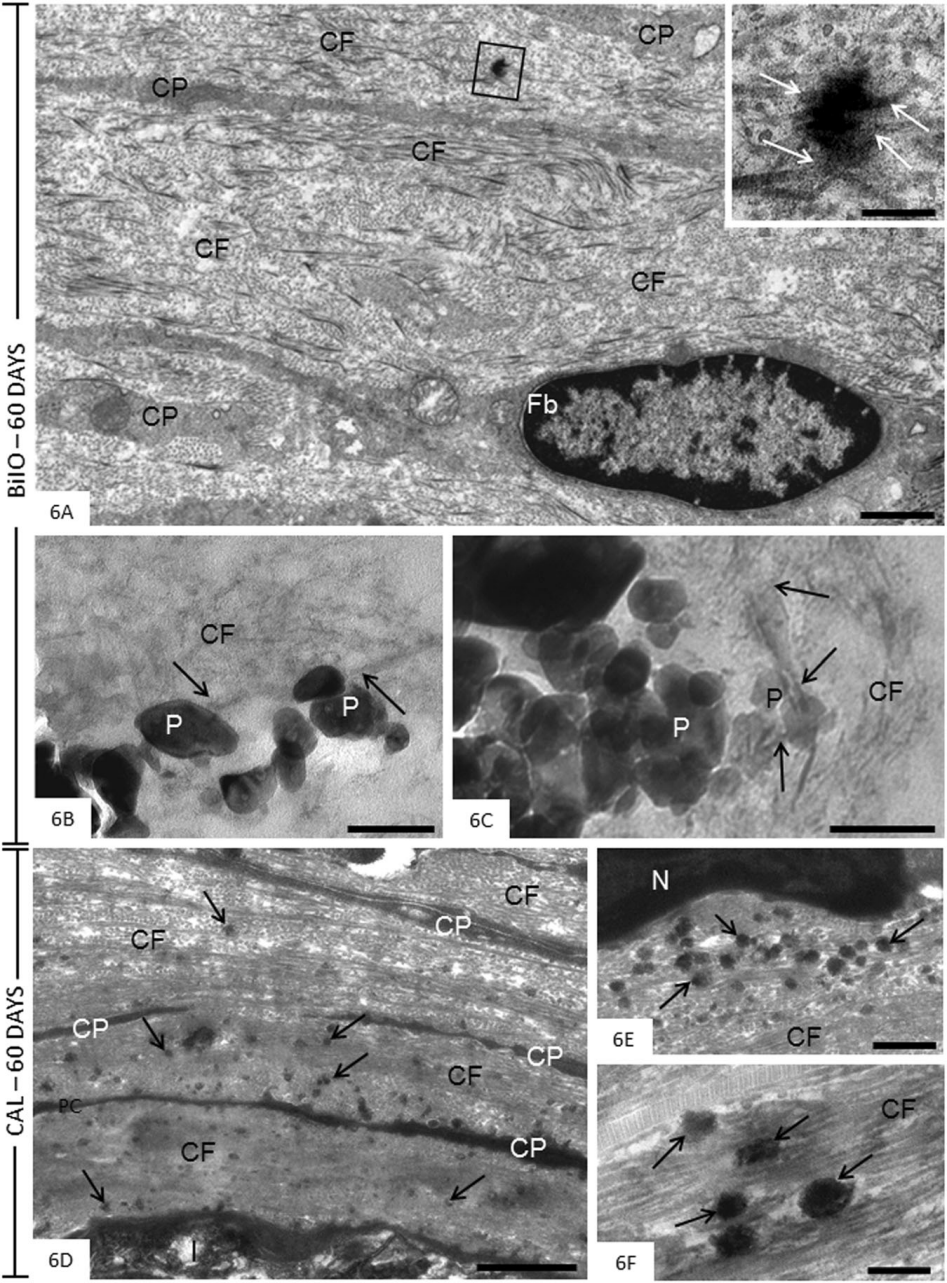
**A–F** Electron micrographs of portions of capsules after 60 days of implantation in the connective tissue of the subcutaneous. **A–C** (BIO specimens) The Fig. 6A shows an electron-opaque structure (outlined area) in the collagen-rich extracellular matrix. The inset, high magnification of outlined area, reveals that some collagen fibrils are in continuity with this electron-opaque structure (arrows). Fb, fibroblast; CP, cytoplasmic processes; CF, collagen fibrils. In (**B**, **C**), the BIO particles (P) are surrounded by thin collagen fibrils (CF); some fibrils (arrows) appear to be in close juxtaposition to the material particles (P). Bars: 2.5 μm (Fig. 6A), 0.5 μm (Fig. 6A, inset) and 200 nm (Fig. 6B, C). **D–F** (CAL specimens). In the Fig. 6D, a portion of capsule exhibits bundles of densely packed collagen (CF) and thin cytoplasmic processes (CP) adjacent to the opening of the tube (I) implanted in the subcutaneous connective tissue. Several electron-opaque round/ovoid shaped structures (arrows) are dispersed by rich-fibrous matrix. Figure 6E and F—high magnification of portions of capsules exhibiting electron-opaque structures (arrows). Round, ovoid or irregularly shaped structures (arrows) with variable electron-opacity are observed amongst collagen fibrils (CF). N, nucleus. Bars: 2 μm (Fig. 6D), 1 μm (Fig. 6E) and 500 nm (Fig. 6F)

After 60 days, the capsules around the BIO specimens showed elliptical fibroblasts surrounded by densely packed collagen fibrils bundles (Fig. 6A). Small irregular and electron-opaque structures were observed within these collagen bundles. Some collagen fibrils appeared to be continuous with these electron-opaque structures (Fig. 6A, inset). Collagen fibrils were also found in close juxtaposition to particles of medication dispersed by capsules around the BIO specimens (Fig. 6B, C). In CAL specimens, the capsules contained predominantly bundles of collagen fibrils intermingled with narrow cytoplasmic processes (Fig. 6D). Several round/ovoid structures showing varied electron-opacity were sparsely distributed among the collagen fibrils (Fig. 6D–F).

## Discussion

A wide variety of substances and medications have been used when the endodontic treatment cannot be completed in a single session, particularly in certain circumstances such as non-vital tooth, chronical apical periodontitis, periapical abscess, endodontic treatment of tooth with incomplete root formation [34, 35]. Among these medicaments and substances are calcium hydroxide-based pastes, calcium hydroxide paste associated with chlorhexidine, triple antibiotic paste, tricresol formalin/formocresol, camphorated parachlorophenol, bioceramic-based pastes and bioactive glass. Although calcium hydroxide pastes have been widely used due to biological properties, it has been demonstrated that these medications lead to weakening of root dentine [35]. Despite the antibacterial efficacy of tricresol formalin/formocresol and camphorated parachlorophenol, these substances cause extensive harmful effects on cells and tissues [34]. As bioactive glass has shown an antimicrobial effect, its use as intracanal medication has been investigated [35]. Considering that bioceramic-based materials exhibit good chemical, physical and biological properties [33, 36, 37] was launched the Bio-C Temp (Angelus, Brazil), as an intracanal medication in an attempted to stimulate the repair of periodontal tissues, including mineralized tissues [35].

In the present study, we evaluated whether the Bio-C Temp and Calen medications could interfere in the serum Ca^+2^ and ALP levels since the calcium concentration and ALP activity are essential for biomineralization [15, 27, 29]. It is known that biomineralization is a complex process that depends on calcium supersaturation in the microenvironment [38]. Although both medications release calcium ions [19, 39], at 7 and 15 days, the highest serum calcium level was observed in the CAL samples in comparison with BIO samples. It is possible that this accentuated calcium level may be, at least in part, due to hydrophilic property of Calen, a calcium hydroxide-based paste [39]. Calcium ions release depends on the material properties, particularly, solubility, network structure and permeability to water [40]. The increase in the serum Ca^+2^ level observed in the CAL samples may be attributed to the greater dissociation of calcium hydroxide in Ca^+2^ ions. Calen releases a higher amount of calcium hydroxide molecules than Bio-C Temp [23]. On the other hand, BIO and CAL medications promoted a significant increase in the serum ALP concentration until the 30^th^ day, indicating that these medications may favor the biomineralization. ALP is responsible for release of inorganic phosphate in alkaline microenvironment and is highly expressed during the formation of mineralized tissues. Thus, this enzyme is considered as a marker for osteogenic activity [41].

Considering the increase in the serum ALP concentration, we investigated whether the intracanal medications would present bioactive potential in the connective tissue of rat subcutaneous, a widely used methodology [9, 24, 26, 32, 36, 37]. The morphological and morphometrical analyses of capsules around the medications revealed a significant increase in the number of fibroblasts concomitant to the formation of collagen fibers, indicating that cellular and extracellular matrix damage is reduced over time. The accentuated number of fibroblasts observed in the capsules formed in response to the empty tubes – used as control – compared with BIO and CAL is due to the irritant potential initially promoted in the connective tissue by these medications [24]. Although the implantation of polyethylene tubes into subcutaneous induces an inflammatory reaction in consequence to the surgical trauma [26, 42], the materials placed within tubes release molecules that may initially promote a delay in the rearrangement of the connective tissue [26, 36, 37, 42]. Despite the inflammatory reaction initially caused by Bio-C Temp and Calen [24], the increase in the number of fibroblasts in the capsules over time reinforced that concept that these intracanal medications are biocompatible.

The rearrangement of capsules around the BIO and CAL medications were accompanied by a significant increase in the number of OCN- and OPN-immunolabelled cells over time, indicating that these medications stimulated the cells to produce these proteins. OCN and OPN are non-collagenous proteins of mineralized tissue matrix and, therefore, are markers of cells associated to mineralized tissues [43]. OCN acts in the biomineralization and homeostasis [43] and OPN modulates the adhesion, migration and differentiation of odontoblast progenitor cells, and participates in the tissue repair process [44]. It has been suggested that the beginning of the biomineralization is correlated with the increased release of calcium ions according to OPN expression [45]. Considering that no immunoreactivity was detected in the CG specimens for both proteins, it is conceivable to assume that Bio-C Temp and Calen medications induced the production of osteogenic proteins in the cells of the subcutaneous connective tissue.

Although calcium hydroxide is not present in the composition of Bio-C Temp, the calcium silicate-based materials induce the mineralization due to the formation of calcium silicate gel and calcium hydroxide, in consequence, to its hydration [25, 28], releasing calcium and hydroxyl ions, and increasing the pH of the medium [19]. After 7 days, there is evidence showing that Bio-C Temp provides pH around 10.79 [19]. The alkaline pH and calcium ions stimulate the repair by deposition of mineralized tissue [2]. However, the CAL paste has polyethylene glycol in the vehicle, which has a high molecular weight that allows a slow release of calcium and hydroxyl ions, maintaining its action for a longer time. It has been suggested that when calcium hydroxide is associated with a viscous vehicle, the release of calcium ions can occur for longer period [2]. Here, no significant alteration in the serum calcium levels promoted by Calen (calcium hydroxide-based paste) was seen at 30 and 60 days, indicating that the calcium released is not able to cause changes in serum levels. It is feasible that calcium and hydroxyl ions released for a long time may be responsible for enhanced immunoexpression of OCN and OPN observed in the calcium hydroxide-based paste specimens at 60 days. However, it is important emphasize that, from 30 to 60 days, a significant increase in the immunoexpression for OCN and OPN was also observed in BIO specimens, pointing to the bioactive potential of this bioceramic medication.

At all periods, von Kossa-positive structures, indicative of mineral precipitation, were observed in the capsules around BIO and CAL specimens. Birefringent structures were also seen in the capsules from unstained sections, suggesting the deposition of amorphous calcite [15, 31, 32, 46]. It is known that mineral deposits revealed by von Kossa histochemical reaction as well as birefringent calcite are originated from chemical composition of materials [1]. Furthermore, the presence of round/ovoid electron-opaque structures in the capsules around the BIO and CAL specimens after 60 days of implantation is compatible with the interpretation that these structures may represent globules/nodules of mineral deposition in the collagen-rich matrix [47–49]. Collagen fibrils appeared to protrude from these mineralization globules indicating that mineralized deposits are spreading into the surrounding matrix and collagen fibrils are involved in this process. Our findings point to an interaction of BIO particles with extracellular matrix components, since collagen fibrils in close contact and/or proximity with particles of this medication were observed in ultrastructural analysis suggesting an interaction between medication and tissue components, particularly collagen fibrils. Considering that the elevated concentration of calcium ions is essential in the biomineralization process, the presence of von Kossa-positive structures and birefringent calcite deposits suggest that these medications present bioactive potential [23, 27, 28]. This idea is reinforced by the fact that either von Kossa-positive structures, or birefringent deposits or mineralization globules were not observed in the capsules of CG specimens. These findings are in accordance with other studies carried out in subcutaneous tissues evaluating a calcium hydroxide-based paste [28, 40] and calcium silicate endodontic sealers [14, 15, 26, 31, 32]. Thus, our results suggest that bioceramic intracanal medication (Bio-C Temp) has bioactive potential.

Biomineralization is one important challenge facing endodontics [50]. Thus, there is a consensus that an endodontic material may be bioactive to promote tissue connective repair and hard tissue-like formation [51]. The bioactive potential of endodontic material is an important property to achieve biological sealing [52] and periapical tissues repair [53]. Bio-C Temp was able to induce the precipitation of mineral structures as detected by von Kossa histochemical method culminating in the calcite amorphous precipitation detected under polarized illumination of unstained sections. Moreover, the bioceramic Bio-C Temp medication also promoted an increase in serum ALP levels and induced the synthesis of osteocalcin and osteopontin, which have an important role in the biomineralization process. It is conceivable to suggest that the presence of ALP, osteocalcin and osteopontin in the connective tissue around the medications may be responsible at least in part by formation of mineralization globules. Therefore, the present study suggests that Bio-C Temp may be as effective as Calen paste in inducing tissue and bone repair during endodontic treatment. In addition to apexification, it is suggested that the use of bioceramic intracanal medication emerges as a biological alternative to prevent and treat apical periodontitis. However, more studies are needed, such as clinical trials to guarantee the superiority of bioceramic medication compared to calcium hydroxide in cases of bone repair, considering the time factor and number of delayed dressing changes.

## Conclusion

In the present study, Bio-C Temp, likewise Calen, caused an increase in the levels of ALP, an essential enzyme for biomineralization process, and induced the production of osteocalcin and osteopontin by subcutaneous connective tissue cells as well as the deposition of calcite, suggesting that Bio-C Temp has bioactive potential.

## Supplementary Information

Supplementary material

## Data Availability

The datasets used and/or analysed during the current study available from the corresponding author on reasonable request.
