# Supplementary material for: Bioactive potential of Bio-C Temp demonstrated by systemic mineralization markers and immunoexpression of bone proteins in the rat connective tissue
Source: J Mater Sci Mater Med. 2024 Feb 14;35(1):13. doi: 10.1007/s10856-024-06781-3 (PMC10867037; doi:10.1007/s10856-024-06781-3)
Supplement: Supplementary file 1 — Supplementary material [file 10856_2024_6781_MOESM1_ESM.docx]

**Supplementary material**

**Table 1-** Intracanal medications and chemical composition

| **Intracanal medications** | **Composition** **Chemical composition** |
| --- | --- |
| Calen*  BIO-C TEMP** | Calcium hydroxide + zinc oxide + polyethylene glycol 400 + colophonya  Calcium silicates + calcium aluminate + calcium oxide + calcium tungstate + titanium oxide + base resin |

*SS. White Art. Dent. Ltda, RJ, Brazil, ** Angelus, Londrina, Brazil
